# Hidden Xyloglucan Architecture of the Pollen Intine in *Gagea lutea* Revealed by Sequential Enzymatic Unmasking

**DOI:** 10.3390/biology15030243

**Published:** 2026-01-28

**Authors:** Małgorzata Kapusta, Magdalena Narajczyk, Bartosz J. Płachno

**Affiliations:** 1 Bioimaging Laboratory, Faculty of Biology, University of Gdańsk, 59 Wita Stwosza St., 80-308 Gdansk, Poland; magdalena.narajczyk@ug.edu.pl; 2 Institute of Botany, Faculty of Biology, Jagiellonian University in Kraków, 9 Gronostajowa St., 30-387 Krakow, Poland; bartosz.plachno@uj.edu.pl

**Keywords:** endo-β-mannanase, *Gagea lutea*, hemicellulose, heteromannan, heteroxylan, immunolocalisation, pectate lyase, pollen intine, sequential enzymatic digestion, xyloglucan

## Abstract

The organisation of hemicelluloses within the pollen intine of many monocots remains inadequately characterised, partly due to the masking of epitopes within complex wall matrices. In this study, mature pollen grains of *Gagea lutea* (L.) Ker-Gawl. were analysed using immunofluorescence and immunogold technique with a variety of monoclonal antibodies that target xyloglucan (LM15, LM24, LM25, CCRC-M48), heteroxylan (LM10, LM11), heteromannan (LM21, LM22), and xylan (CCRC-M138). Semithin sections of LR White were examined both untreated and following a sequential enzymatic pretreatment, which included alkaline de-esterification followed by treatment with pectate lyase (RbPel1A) and endo-β-mannanase 5A. In untreated pollen, xyloglucan-related epitopes were identified within the intine, accompanied by additional intracellular labelling for LM15, and LM25; while for LM24 signal was only to the intine ring. Conversely, CCRC-M48 exhibited a more punctate distribution. Neither xylan- nor mannan-related epitopes were detected in the wall or intracellularly. The enzymatic digestion significantly altered the detectability of epitopes, resulting in an increase in continuous wall labelling within the intine across multiple probes. These findings indicate that enzymatic modification of pectic and mannan components has a considerable impact on the apparent distribution of hemicellulose epitopes within the pollen wall of *G. lutea*. Together, these results expand the still limited in situ immunolocalisation evidence base for hemicellulose-related epitopes in pollen, and provide a practical framework for interpreting digestion-dependent changes primarily in terms of epitope accessibility within the intine matrix.

## Simple Summary

The inner pollen wall is essential for hydration and initiation of pollen tube growth, but its molecular organisation is difficult to resolve because many wall components are embedded within the matrix and therefore hard to detect by immunolabelling. Here, we examined mature pollen grains of *Gagea lutea* using fluorescence microscopy and transmission electron microscopy, combined with highly specific antibodies recognising defined polysaccharide epitopes (xyloglucan, heteroxylan, heteromannan and xylan). We compared untreated sections with sections subjected to a stepwise enzymatic treatment (pectate lyase followed by endo-β-mannanase) aimed at removing selected matrix constraints, particularly pectins, and thereby increasing epitope accessibility. Xyloglucan-related epitopes were detected in the intine and became markedly more detectable and more continuous after sequential digestion. Quantitative fluorescence measurements confirmed that the largest increases occurred only after the combined digestion steps, supporting the interpretation that the treatment primarily enhanced epitope accessibility rather than increasing polymer abundance. Several other epitopes remained below the detection threshold under the conditions used; therefore, non-labelling should be interpreted as non-detection rather than definitive absence. Overall, these results provide an improved framework for mapping concealed wall architecture in monocot pollen.

## 1. Introduction

Hemicelluloses are structurally diverse polysaccharides that play a crucial role in the architecture and functionality of plant cell walls. They can be defined as cell wall polysaccharides that bind strongly to cellulose microfibrils through hydrogen bonds and Van der Waals interactions [1,2,3]. In general, hemicelluloses comprise a heterogeneous group of plant-derived polysaccharides consisting of D-xylose, D-mannose, D-galactose, L-arabinose, and 4-O-methyl-D-glucuronic acid. In pollen grains, their contribution has historically received less attention than that of pectins or cellulose, yet early biochemical analyses already demonstrated their abundance. For example, Amin et al. [4] reported that a hemicellulose fraction isolated from *Phoenix dactylifera* pollen contained arabinose, galactose, xylose, and rhamnose residues. In olive (*Olea europaea*) pollen, comparative analyses of mature versus germinated grains indicated changes in cell-wall-associated polymer fractions, consistent with wall remodelling during germination [5]. Similarly, Nakamura et al. [6] demonstrated that hemicelluloses extracted from *Camellia japonica* pollen tubes are extractable as a hemicellulose fraction composed essentially of xylans and/or glucomannans, and that they can contribute to the developing pollen tube wall. Together, these early datasets support the view that hemicellulose-containing fractions are remodelled during pollen activation and tube emergence, but they do not resolve polymer identity at epitope level. Other cell wall components such as arabinogalactans and the pectin-centered remodelling of pollen walls and pollen-tube cell walls have already been covered extensively in the review literature [7,8,9,10].

Species-specific differences are evident when hemicelluloses are evaluated with genetic and immunological tools. In rice, xylan clustering on the pollen surface is required for correct exine patterning and male fertility [11]. In addition, OsmiR528-dependent regulation has been linked to rice pollen intine formation through effects on a uclacyanin and flavonoid metabolism [12]. In eudicot pollen, developmental timelines integrating histology, TEM/SEM, and immunolabelling have demonstrated sequential deposition and remodelling of wall polymers during pollen maturation (shown in tomato) [13]. In seagrasses, pollen wall traits are strongly modified in association with hydrophilous pollination, which shifts functional emphasis towards the inner wall during transport and germination even when exine features are reduced [14]. Biophysical work with cellulose–hemicellulose hydrogel systems supports the concept that hemicelluloses modulate fibre–matrix interactions and mechanics in a structure-dependent manner [15]. From an evolutionary perspective, the presence of hemicellulose-active enzymes and associated remodelling capacity has been discussed already in charophyte lineages, supporting deep evolutionary roots for hemicellulose processing [16]. More broadly, the plant cell wall is increasingly conceptualised as a dynamic and adaptable composite, which frames hemicelluloses as active determinants of wall function rather than passive fillers [17]. Finally, environmental drivers relevant for pollen biology, including climate-linked changes in pollen exposure and allergic disease burden, provide applied motivation for understanding polymer-level determinants of wall resilience and release [18,19].

Pectic homogalacturonan can mask xyloglucan epitopes in situ, complicating direct immunodetection and interpretation [20]. Therefore, antibody-based localisation benefits from defined epitope knowledge, and controlled unmasking strategies. High-resolution oligosaccharide microarrays provide a framework for validating antibody specificities and interpreting localisation patterns [21]. In addition, mannan epitopes can be masked in intact walls, again influencing detectability without necessarily reflecting true absence [18]. For xylans, LM10 and LM11 are widely used probes, and their binding preferences have been characterised using xylan/arabinoxylan oligosaccharide panels and synthetic glycan arrays [22,23]. Glycome profiling toolkits further expand coverage across hemicellulose classes and enable multi-epitope comparisons [24,25]. For the CCRC panel used here, CCRC-M48 has been used as a probe recognising nonfucosylated xyloglucan motifs in grass/eudicot comparisons and related biochemical contexts [26], whereas CCRC-M138 has been applied as a xylan-directed antibody in studies of secondary wall xylan organisation [27]. Against this background, the aim of this study is to map the localisation of selected hemicellulose-related epitopes in mature pollen grain and to test how sequential enzymatic unmasking modifies the detectability of these epitopes in the pollen wall and intracellular compartments.

Beyond their chemical diversity, xyloglucans in pollen walls are functionally important in maintaining the mechanical balance between rigidity and flexibility during hydration and germination [28,29,30]. In parallel, callose (a β-1,3-glucan, distinct from xyloglucan) provide dynamic reinforcement and remodelling capacity, enabling pollen tubes to withstand high turgor pressure while directing polarised growth [10,31]. Despite their importance, most research to date has focused on pectins and callose, leaving the roles of xyloglucans in pollen walls comparatively underexplored [10,16]. This knowledge gap is particularly evident for monocot pollen [11], where mannans and xylans have been reported, but their localisation and functional dynamics remain poorly understood. Furthermore, previous biochemical studies did not allow for in situ resolution of hemicellulose distribution, which can now be addressed through immunolocalisation using monoclonal antibodies combined with sequential enzymatic unmasking [20,22,32].

The aim of this study is to provide the first comprehensive survey of hemicellulose epitopes in pollen walls of a monocot species, using an extended panel of monoclonal antibodies and to demonstrate how sequential enzymatic digestion with pectate lyase and endo-β-mannanase can reveal masked epitopes, offering novel insights into the distribution and remodelling of hemicelluloses in mature pollen grain. In this study, we also aimed to determine how these wall components are spatially organised in mature pollen grain and whether they are also detectable within the apoplastic interface between the pollen grain plasma membrane (PGPM) and the generative cell plasma membrane (GC PM), analogous to the AGP-enriched domain previously reported for this species [33]. Finally, because environmental stress and climate change are increasingly recognised as factors altering pollen wall composition and thickness, with implications for allergenicity [34,35,36], understanding the dynamics of xyloglucan deposition is not only relevant for reproductive biology but may also contribute to predicting how changing environments shape pollen allergenic potential. In this study, treatment-dependent changes in labelling intensity and pattern following alkaline de-esterification and enzymatic digestion are interpreted primarily as increased epitope accessibility (unmasking) within the wall matrix, rather than as a direct increase in the abundance of the corresponding polymers.

## 2. Materials and Methods

### 2.1. Plant Material

Flowers at anthesis of *Gagea lutea* (L.) Ker Gawl. were collected from selected sites of Steffens Park—a park near the city centre in Gdańsk (Poland), in March–April 2023 and 2024. Pollen was collected from at least 20 individual plants, pooled and thoroughly mixed prior to dehydration and freezing. For qualitative and quantitative analyses, interpretation was based on pellets containing approximately 300–600 pollen grains.

### 2.2. Immunofluorescence

Pollen grains were first rehydrated and activated in Read’s germination medium [37], then fixed overnight at 4 °C in 1.6% (*w*/*v*) paraformaldehyde and 2% (*v*/*v*) glutaraldehyde in 50 mM PIPES (Sigma Aldrich, Poznań, Poland) buffer (pH 6.8) containing 10 mM EGTA (Sigma Aldrich, Poznań, Poland) and 1 mM MgCl_2_ (Sigma Aldrich, Poznań, Poland) [33]. Samples were post-fixed for 15 min in 0.5% osmium tetroxide, dehydrated through an acetone series and embedded in LR White acrylic resin (Microshop, Laszczki, Poland). Semi-thin sections (0.3–0.5 µm) were cut using a Leica Ultracut UCT ultramicrotome (Leica Microsystems, Wetzlar, Germany). and mounted on glass slides. Sections were rehydrated in PBS and blocked with 1% (*w*/*v*) bovine serum albumin (BSA-c; Aurion, Wageningen, The Netherlands) for 15 min. Primary antibodies were applied overnight at 4 °C in a humid chamber. To analyse the spatial distribution of hemicelluloses in mature pollen grains, monoclonal antibodies recognising epitopes of xyloglucans, heteromannans and heteroxylans were used. The following antibodies were applied: LM10, LM11 [20,22,24], LM15 [20], LM21, LM22 [22], LM24 [21], LM25 [21] (PlantProbes, Leeds, UK) and CCRC-M48, CCRC-M138 [25] (Complex Carbohydrate Research Center, University of Georgia, Athens, GA, USA). All antibodies were diluted 1:20 in phosphate-buffered saline (PBS, pH 7.2). After rinsing with PBS, slides were incubated for 2 h with FITC-conjugated or AlexaFluor™ 488-conjugated secondary antibodies (goat anti-rat or anti-mouse, Abcam, Cambridge, UK; 1:100 dilution). Untreated sections were counterstained with propidium iodide [PI (Sigma Aldrich, Poznań, Poland); final concentration 2.5 µg/mL in PBS] to visualise nuclei and provide a red reference signal for the sporoderm outline during imaging. Samples were mounted in Mowiol^®^ 4-88 (Sigma Aldrich, Poznań, Poland) containing 2.5% 1,4-diazabicyclo[2.2.2]octane (DABCO; Roth, Zielona Góra, Poland) to reduce photobleaching. Negative controls were prepared by omitting the primary antibody [38]. Imaging was performed using a Leica STELLARIS 5 WLL confocal microscope (gain was set to 18.4, and the laser intensity was set to 13.9%) equipped with Lightning deconvolution (Leica Microsystems, Wetzlar, Germany). Cellulose and xyloglucan were labelled using Carbotrace 540 [Ebba Biotech AB, Nobels väg 16 S-171 65 Solna, Sweden; https://www.ebbabiotech.com/products/carbotrace-680?variant=47885141180748 (accessed on 14 December 2025)]. Laser power, detector gain and pinhole size were kept constant for all replicates. Images were acquired as maximum projection from taken Z-stacks.

### 2.3. Immunogold

For immunogold reactions, rehydrated pollen grains were fixed as for immunofluorescence analysis but embedded in Epon resin (Sigma Aldrich, Poznań, Poland). Immunogold reactions were conducted utilising the LM15, LM24, LM25 and CCRC-M48 primary antibodies at a dilution of 1:10, incubated overnight at 4 °C and with secondary antibodies (goat anti-rat or anti-mouse) conjugated to 10 nm colloidal gold particles (1:50, Sigma-Aldrich, Poznań, Poland) for a duration of 3 h at room temperature. The sections were stained with Lead citrate (Microshop, Laszczki, Poland) and URANYLess (Microshop, Laszczki, Poland) [38,39] subsequently analysed using an FEI Tecnai Spirit BioTWIN (Thermo Fisher Scientific, Warszawa, Poland) transmission electron microscope, located at the Bioimaging Laboratory within the Faculty of Biology at the University of Gdańsk [33]. Negative controls for all immunogold were created by omitting the primary antibody step.

### 2.4. Enzymatic Pretreatments

To selectively remove homogalacturonan and mannans, semithin sections embedded in LR White were subjected to a two-step enzymatic digestion. For de-esterification of pectic homogalacturonan, the sections were first incubated in 0.1 M sodium carbonate [Na_2_CO_3_ (Sigma Aldrich, Poznań, Poland), pH 11.4] for 2 h at room temperature, followed by three rinses (5 min each) in distilled water and 50 mM CAPS buffer (pH 10.0). The samples were subsequently digested with RbPel1A pectate lyase (NZYTech, CH1090, Lisbon, Portugal) at a final concentration of 10 µg mL^−1^ for 2 h at room temperature in a solution containing 50 mM *N*-cyclohexyl-3-aminopropane-sulfonic acid (CAPS, Sigma-Aldrich Sp. z o.o., Poznań, Poland) and 2 mM CaCl_2_ (Sigma-Aldrich, Poznań, Poland). The enzyme stock (0.5 mg mL^−1^) was freshly diluted immediately before use and the reaction was stopped by washing the samples three times in buffer. To remove hemicellulosic mannans, the sections were subsequently incubated with endo-β-mannanase 5A (NZYTech, MB27501, Lisbon, Portugal) prepared in 50 mM PIPES buffer (piperazine-*N*,*N*′-bis [2-ethanesulfonic acid], Sigma-Aldrich, Poznań, Poland) at pH 6.8, at a final enzyme concentration of 10 µg mL^−1^ for 2 h at room temperature. No Ca^2+^ was added to the endo-β-mannanase buffer. Following digestion, the samples were rinsed three times in the same buffer and transferred directly to the blocking step prior to immunolabelling. Negative control reactions were processed in parallel without enzyme addition. All enzymatic treatments were performed on the same day as antibody labelling to ensure maximal enzyme activity and preservation of antigenic epitopes.

### 2.5. Statistical Analysis

For quantitative analysis, single optical sections were acquired using a confocal microscope (detector gain 4.8; laser intensity 13.9%). Corrected mean fluorescence intensity (MFI_corrected, a.u.) was quantified from 2D freehand regions of interest (ROIs) manually drawn over the intine in single optical sections. All images used for quantification were acquired using identical confocal settings (13.9%, 4.8%, 1 AU, 400 Hz, zoom = 5.0) to allow for direct comparison of intensity values across conditions. Two independent imaging runs (run 1 and run 2) were analysed. For each antibody × treatment combination, 10 pollen grains per run were measured (n = 20 grains per combination; total N = 240 measurements across 4 antibodies × 3 treatments × 2 runs).

Statistical analyses were performed in Statistica v13.3 (TIBCO). A factorial general linear model (GLM) ANOVA was applied with antibody (4 levels) and digestion treatment (3 levels) as fixed factors and run (2 levels) included as a run/blocking factor; all two-way and three-way interactions were included. The main effect of run was not significant (F(1,216) = 2.503, *p* = 0.115), whereas antibody (F(3,216) = 149.502, *p* < 0.000001) and treatment (F(2,216) = 77.303, *p* < 0.000001) were significant, as was the antibody × treatment interaction (F(6,216) = 4.700, *p* = 0.000161). Post hoc multiple comparisons were performed using Tukey’s HSD (family-wise alpha = 0.05) across all antibody × treatment combinations (12 groups).

## 3. Results

### 3.1. Untreated Mature Pollen Grains

**Figure 1:**
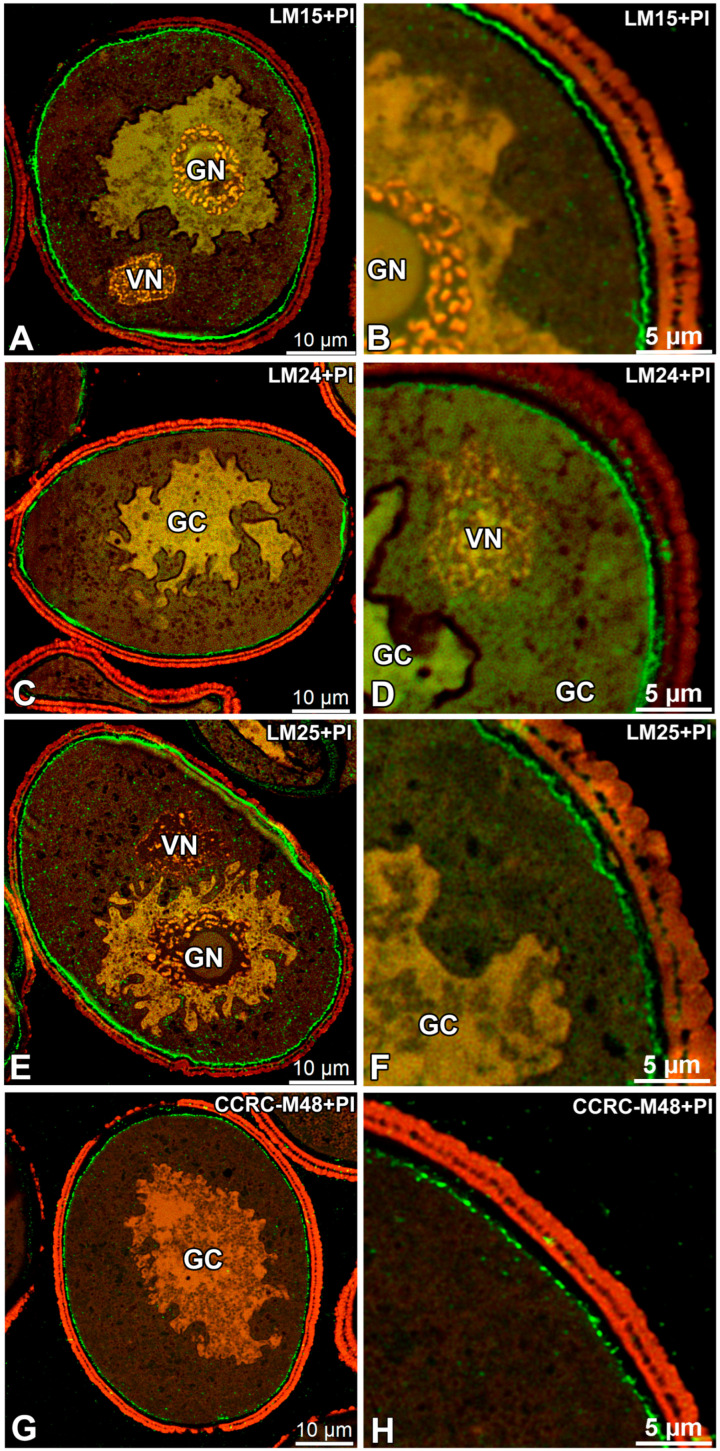
Localisation of cell-wall epitopes recognised by LM15, LM24, LM25 and CCRC-M48 in mature pollen grains of *Gagea lutea*. (**A**,**B**) LM15; (**C**,**D**) LM24; (**E**,**F**) LM25; (**G**,**H**) CCRC-M48. Confocal immunofluorescence images show the antibody signal (green) in overview (**A**,**C**,**E**,**G**) and higher magnification of the pollen periphery (**B**,**D**,**F**,**H**). Propidium iodide (PI, red fluorescence) was used as a counterstain to localise nuclei. (GC)—generative cell; (GN)—generative nucleus; (VN)—vegetative nucleus. Scale bars: 10 µm (**A**,**C**,**E**,**G**); 5 µm (**B**,**D**,**F**,**H**).

**Figure 2:**
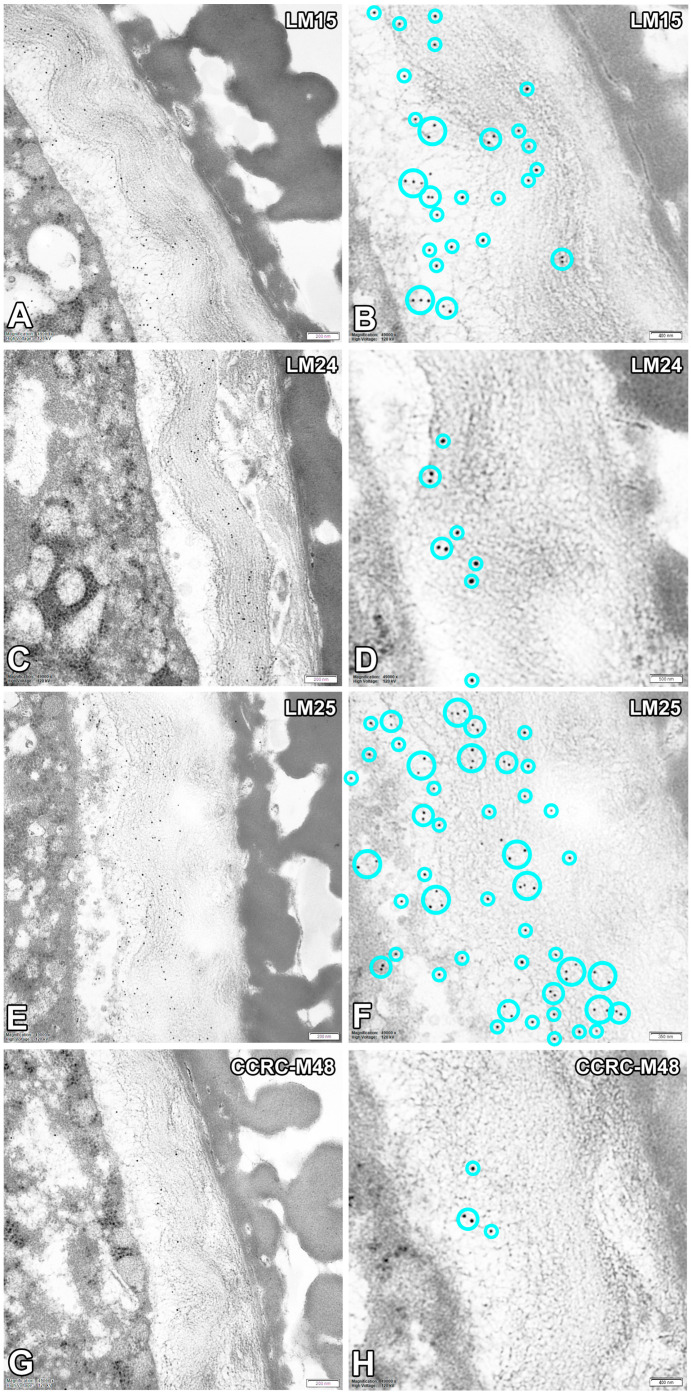
Immunogold localisation of cell-wall epitopes recognised by LM15, LM24, LM25 and CCRC-M48 in mature pollen grains of *Gagea lutea*. (**A**,**B**) LM15; (**C**,**D**) LM24; (**E**,**F**) LM25; (**G**,**H**) CCRC-M48. Immunogold images show the antibody signal (gold particles) in overview (**A**,**C**,**E**,**G**) and higher magnification of the pollen intine (**B**,**D**,**F**,**H**). Representative gold particles were marked with a light cyan circle for better clarity. Scale bars: 200 nm (**A**,**C**,**E**,**G**); 400 nm (**B**,**H**); 500 nm (**D**); 350 nm (**F**).

In untreated mature pollen grains of *G. lutea*, LM15, LM24, LM25 and CCRC-M48 epitopes were detected in the pollen wall, with additional intracellular signal for LM15 (Figure 1A,B and Figure 2A,B) and LM25 (Figure 1C,D and Figure 2C,D). LM15 labelling was distinct at the pollen periphery in the intine and, within the vegetative cell, appeared as small punctate structures in the cytoplasm (Figure 1A–C). LM24 signal was present with moderate wall-associated fluorescence restricted to the intine (Figure 1E,F and Figure 2E,F). LM25 produced a strong wall outline and numerous punctate signals in the vegetative cell cytoplasm (Figure 1C,D). In contrast, CCRC-M48 binding was confined to the intine domain, where punctate labelling was observed (Figure 1G,H and Figure 2G,H). For all immunogold-labelled preparations, ultrastructural observations revealed 10 nm gold particles (electron-dense dots) associated mainly with the intine domain (Figure 2A–H).

**Figure 3:**
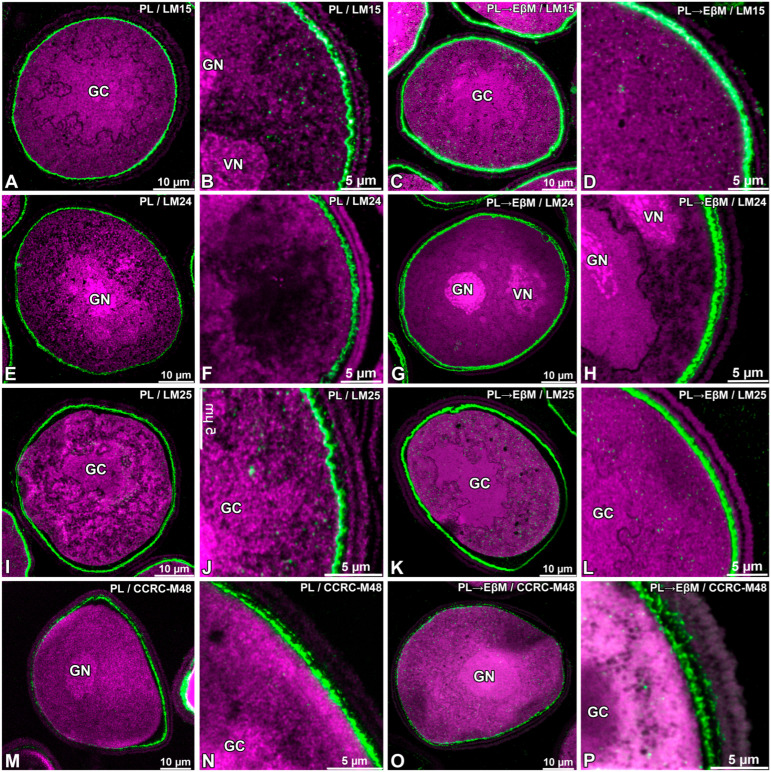
Effect of enzymatic digestion on the localisation of epitopes recognised by LM15, LM24, LM25 and CCRC-M48 in mature pollen grains of *Gagea lutea*. (**A**–**D**) LM15; (**E**–**H**) LM24; (**I**–**L**) LM25; (**M**–**P**) CCRC-M48. Sections were treated with pectate lyase (PL) alone (**A**,**B**,**E**,**F**,**I**,**J**,**M**,**N**) or sequentially with pectate lyase followed by endo-β-mannanase (PL → EβM) (**C**,**D**,**G**,**H**,**K**,**L**,**O,P**) prior to immunolabelling. Antibody signal is shown in green fluorescence; magenta indicates autofluorescence. (GC)—generative cell; (GN)—generative nucleus; (VN)—vegetative nucleus.

In the same untreated material, LM10, LM11, LM21, LM22 and CCRC-M138 did not show any detectable signal in the pollen wall or within the cytoplasm (Figure 3A,D,G,J,M).

### 3.2. Enzymatic Digestion

Following treatment with pectate lyase (PL), notable alterations in the distribution of the LM15, LM24, LM25 and CCRC-M48 epitopes were documented (Figure 3A,B,E,F,I,J,M,N). LM15 showed an increased wall-associated signal in the pollen wall, while the intracellular punctate signal observed in untreated samples remained evident (Figure 3B). The fluorescence of LM24 was diminished within the cytoplasm (Figure 3E,F), whereas the signal within the intine was strongly pronounced. LM25 maintained a wall-associated signal, with intracellular labelling still observable (Figure 3J). The fluorescence of CCRC-M48 was restricted to the intine region of the wall; relative to untreated samples, PL treatment converted the intine-associated signal into a continuous peripheral ring (Figure 3M,N).

Following sequential digestion with pectate lyase and endo-β-mannanase (PL followed by EβM), LM15, LM24 and LM25 signals within the intine were observed as a continuous ring (Figure 3C,D,G,H,K,L). In the cytoplasm, intracellular fluorescence was still observed and the intine showed strong continuous signal (Figure 3C,D,G,H,K,L). CCRC-M48 signal persisted in the intine but became less continuous (more discontinuous/patchy) compared with PL-only treatment (Figure 3O,P).

**Figure 4:**
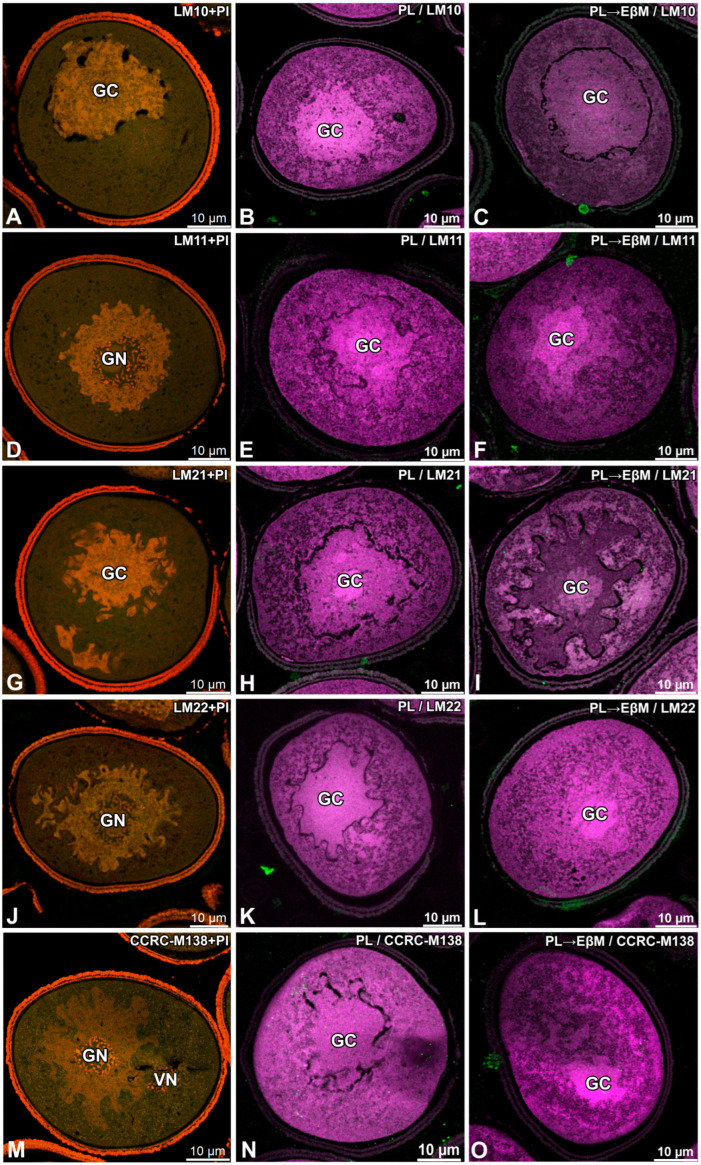
Effect of enzymatic digestion on the localisation of epitopes recognised by LM10, LM11, LM21, LM22 and CCRC-M138 in mature pollen grains of *Gagea lutea*. (**A**–**C**) LM10; (**D**–**F**) LM11; (**G**–**I**) LM21; (**J**–**L**) LM22; (**M**–**O**) CCRC-M138. Untreated sections counterstained with propidium iodide (PI) are shown in the left column (**A**,**D**,**G**,**J**,**M**). Sections were treated with pectate lyase (PL) alone (**B**,**E**,**H**,**K**,**N**) or sequentially with pectate lyase followed by endo-β-mannanase (PL → EβM) (**C**,**F**,**I**,**L**,**O**) prior to immunolabelling. Antibody signal is shown in green fluorescence; PI is red; magenta indicates autofluorescence. (GC)—generative cell; (GN)—generative nucleus; (VN)—vegetative nucleus.

For the second antibody set, digestion effects did not show any evident changes in signal. After PL treatment and PL followed by EβM, the lack of signal in the intine persisted across all used antibodies and distinct intracellular labelling was still not apparent (Figure 4C,F,I,L,O).

No signal from the sporoderm was detected in all conducted immunolabellings. In addition, no specific signal was detected at the interface between the pollen grain cell membrane (PGCM) and the generative cell plasma membrane (GC PM) under any labelling condition (Figure 1, Figure 2, Figure 3 and Figure 4).

### 3.3. Quantification of Intine Fluorescence (MFI)

**Figure 5:**
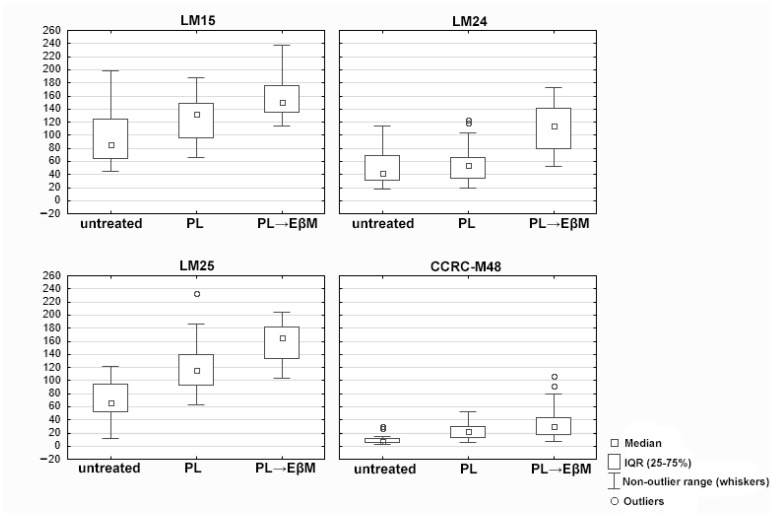
Post hoc multiple comparisons (Tukey’s HSD) for treatment effects within each antibody (LM15, LM24, LM25, CCRC-M48). Adjusted *p*-values (family-wise alpha = 0.05).

Sequential digestion (pectate lyase followed by endo-β-mannanase (Table 1 and Figure 5; Tables S1 and S2) significantly increased the intine signal for LM15, LM24 and LM25, compared with untreated pollen (Tukey’s HSD, adjusted *p* = 0.000018 for each antibody), whereas pectate lyase alone did not produce a significant change for these antibodies (LM15: adjusted *p* = 0.293848; LM24: adjusted *p* = 0.999113; LM25: adjusted *p* = 0.999998). For LM25, the signal after sequential digestion was also significantly higher than after pectate lyase alone (adjusted *p* = 0.002655). In contrast, CCRC-M48 showed no statistically significant change in signal intensity after either pectate lyase or sequential digestion relative to untreated pollen (adjusted *p* = 0.967402 and *p* = 0.144472, respectively).

### 3.4. Control Reactions

Carbotrace 540 staining of hydrated, non-germinating pollen grains yielded a distinct signal that was confined to the pollen wall domain, delineating the inner surface of the exine and forming a continuous ring within the intine (Supplementary Figure S1A). This signal was particularly observed in the region of the germination aperture, where a brighter, crescent-shaped accumulation was observed. No fluorescence from Carbotrace 540 was detected within the cytoplasm of either vegetative or generative compartments. Propidium iodide (PI) counterstaining effectively localised the nuclei and provided contrast within the cytoplasm.

Negative controls supported the specificity of the immunolabelling. In immunogold TEM controls performed without the primary antibody, 10 nm gold-labelled secondary antibodies did not produce specific labelling of the wall domain (Figure 4B,C). In immunofluorescence controls omitting the primary antibody, FITC-conjugated or AlexaFluor™ 488-conjugated secondary antibodies did not generate specific green fluorescence in untreated sections or after PL or PL followed by EβM digestion (Supplementary Figure S1D–I).

## 4. Discussion

Primary cell walls differ substantially between major Angiosperm lineages, with grasses and other monocots often described as having type II wall features, including a higher prominence of heteroxylans and mixed-linkage glucans and a different quantitative balance of pectins and xyloglucans relative to many eudicot primary walls [1,2,40,41,42,43]. Against this background, our immunolabelling data in mature pollen grains of *Gagea lutea* indicate that xyloglucan-related epitopes (LM15, LM24, LM25) are prominent in the intine, while xylan- and mannan-related epitopes remained below the detection threshold under the applied conditions [1].

A consistent feature across markers was the strong association of gold particles with the intine domain in immunogold TEM, supporting the fluorescence-based localisation and reinforcing the conclusion that the intine is the principal site of these detected carbohydrate epitopes in *G. lutea* pollen. In addition to wall-associated signals, punctate intracellular labelling was detected for LM15 and LM25. The most straightforward interpretation of this intracellular punctate signal is that it marks compartments involved in the biosynthesis, processing and/or trafficking of non-cellulosic cell wall polysaccharides prior to their deposition, consistent with the established Golgi/endomembrane localisation of hemicellulose synthesis and secretion pathways [1].

Carbotrace 540 staining showed fluorescence primarily restricted to the intine and was enriched at the aperture region, with no cytoplasmic signal. According to the manufacturer’s characterisation, Carbotrace 540 is an optotracer designed to label repetitive motifs in proteins and carbohydrates; labelling is typically strongest for protein aggregates, homoglucans such as cellulose and has shown to bind to the hemicellulose xyloglucan (Ebba Biotech AB, product information). In studied pollen sections, Carbotrace 540 therefore supports the presence of a glucan-rich, wall-localised polymeric network in the intine and aperture region, consistent with a cellulose-containing intine matrix and showed similar signal as Carbotrace 680 [33].

Sequential pretreatments (alkaline de-esterification followed by pectate lyase and endo-β-mannanase) increased the continuity and apparent accessibility of wall-associated fluorescence for several probes, particularly in the intine. Because intracellular fluorescence remained detectable after digestion in our dataset, the results support an unmasking model (improved wall epitope accessibility) rather than a “loss of cytoplasmic signal” model. Mechanistically, this is consistent with enzymatic and chemical modification of the wall matrix that increases antibody access to epitopes without necessarily altering intracellular pools [1]. Claims of true intracellular-to-wall redistribution would require evidence beyond the current imaging readout, such as quantitative co-localisation across conditions or biochemical detection of released oligosaccharides. Consistent with this interpretation, cell wall modification has been explicitly highlighted as a factor that can determine antibody epitope accessibility and, in some wall domains, may result in little or no detectable antibody signal [41].

Finally, the enrichment of wall-associated signals in the aperture region is consistent with a structurally specialised intine subdomain at the germination site. A functional interpretation that a glucan-rich intine architecture contributes to rapid, local wall remodelling during germination is a testable hypothesis rather than a direct conclusion from the present localisation data and should be treated as such [42,43]. Ultrastructural work on monocot pollen has resolved the intine into distinct sublayers, typically described as an outer exintine and an inner endintine, with specialised thickening in the aperture region (often referred to as the Zwischenkörper). This architectural context is relevant for interpreting digestion-dependent “unmasking” as changes in accessibility within a stratified intine rather than a uniform bulk wall [44]. Notably, the hemicellulose immunolabelling pattern in *G. lutea* pollen, which does not map cleanly onto “typical dicot” versus “typical monocot” expectations, can be explained by lineage-level differences in primary-wall hemicellulose networks and digestion-dependent epitope masking [1,2]. Primary walls are often discussed as Type I (common in eudicots and many non-commelinid monocots) versus Type II (common in commelinid monocots, especially grasses), with Type I walls being comparatively xyloglucan-rich and Type II walls being enriched in glucuronoarabinoxylans and mixed-linkage glucans [1,2,42,45]. This taxonomic framework is well established and provides a concrete reason why “typical monocot” generalisations (frequently derived from Poaceae) can mispredict epitope behaviour in non-grass monocots [42,45]. In addition, the partial detectability of xyloglucan-related epitopes (LM15, CCRC-M48) prior to digestion and their strong enhancement after mild pectate lyase and endo-β-mannanase treatment, is consistent with pectin-dependent epitope masking and subsequent unmasking rather than an actual increase in polymer amount, as shown experimentally for LM15-recognised xyloglucan epitopes in pectin-rich walls after pectate lyase treatment [20]. Because pectin chemistry in the intine is functionally linked to hydration dynamics and germination competence, including via pectin methylesterase activity, we treat the digestion-sensitive signal increase primarily as a readout of accessibility within a rehydrating, less constrained matrix [46]. In the ecological context, male gametophytes of early-season species can retain function at very low temperatures and in *G. lutea* specifically, heat temperature experiments have been linked to reduced reproductive performance including reduced pollen germination [47,48,49]. On that basis, we suggest that a comparatively more readily rehydrating intine architecture could contribute to rapid hydration and germination under fluctuating early spring conditions [50]. Finally, independent immunocytochemical datasets using overlapping antibody panels show pronounced cell-wall microdomain heterogeneity and epitope-selective non-detection in plant tissues, reinforcing that immunolabelling patterns depend on microdomain context and epitope accessibility [38]. Beyond comparisons and structure, the intine pattern of xyloglucan epitopes may also matter for pollen function. The intine is the hydrated part of the pollen wall that must remodel quickly when pollen activates and the tube starts to grow, and this begins at the aperture [51,52]. In our study, xyloglucan epitopes were clearly detected in the intine and appeared enriched at the aperture. This is consistent with xyloglucan helping to organise and support a cellulose-based wall network, while still allowing for controlled stretching in this small, critical region. Importantly, the effects of the enzyme pre-treatments should be seen as changes in how well the epitopes are exposed (masking and unmasking), not as direct proof that the amount of xyloglucan changed. This matters because epitope exposure is expected to change during hydration and early germination, when wall porosity, charge, and polymer interactions are modified. Therefore, better exposure of xyloglucan epitopes at the aperture may help the wall yield quickly during hydration and support reliable tube emergence under fluctuating environmental conditions [44,51,52].

## 5. Conclusions

1. Sequential pretreatments and enzymatic digestion (alkaline de-esterification followed by pectate lyase and endo-β-mannanase) increased the detectability and continuity of wall-associated signals for several probes. The most straightforward interpretation is that these treatment-dependent changes primarily reflect altered epitope accessibility (unmasking) within the intine matrix, rather than an increase in polymer abundance.
2. Quantitative fluorescence measurements support a treatment-dependent change in epitope accessibility, with the combined digestion producing significant increases in LM15, LM24 and LM25, whereas pectate lyase alone did not.
3. Under the conditions used here, LM10, LM11, LM21, LM22 and CCRC-M138 remained below the detection threshold. Because immunolabelling reports epitope presence and accessibility, negative results cannot be interpreted as definitive absence of the corresponding polymers.
4. CCRC-M48 did not show statistically significant intensity changes after digestion, indicating that different xyloglucan-associated epitopes can respond differently to the same unmasking workflow.

## Figures and Tables

**Table 1:** Summary of post hoc (Tukey’s HSD) comparisons for intine immunolabelling intensity (MFI) after enzymatic digestion: pectate lyase (PL) versus untreated and sequential digestion (PL → endo-β-mannanase, EβM) versus untreated, with a brief interpretation for each antibody.

Antibody	PL vs. Untreated	PL → EβM vs. Untreated	Short Interpretation
LM15	↑ (ns; ***p*** = 0.293848)	↑ (****; ***p*** = 0.000018)	Strongest increase after PL → EβM; PL not significant
LM24	– (ns; ***p*** = 0.999113)	↑ (****; ***p*** = 0.000018)	Increase only after PL → EβM; PL ~ no effect
LM25	– (ns; ***p*** = 0.999998)	↑ (****; ***p*** = 0.000018)	Increase mainly after PL → EβM; PL vs. untreated not significant (but PL → EβM > PL: **; ***p*** = 0.002655)
CCRC-M48	↑ (ns; ***p*** = 0.967402)	↑ (ns; ***p*** = 0.144472)	Upward trend, but no significant differences after Tukey’s adjustment

Upward arrows (↑) indicate an increase in corrected mean fluorescence intensity (MFI) relative to the untreated control, while the dash (–) indicates no change relative to the untreated control. Significance is based on Tukey’s HSD post hoc test following the factorial GLM ANOVA: ns, not significant (*p* ≥ 0.05); ** *p* < 0.01; **** *p* < 0.0001. All *p* values shown are Tukey-adjusted.

## Data Availability

The data presented in this study are available on request from the corresponding author.

## Supplementary Materials

The following supporting information can be downloaded at https://www.mdpi.com/article/10.3390/biology15030243/s1. Figure S1: Control reactions for labelling and enzymatic digestion assays in mature pollen grains of Gagea lutea; Table S1: Corrected mean fluorescence intensity (MFI) measured in the intine ROI. Fixed factors: pool (2 levels), antibody (LM15, LM24, LM25, CCRC-M48) and treatment (untreated, pectate lyase (PL), PL followed by endo-β-mannanase (PL → EβM)). Statistica v13.3 (TIBCO); Table S2: Post hoc multiple comparisons (Tukey’s HSD).
