# Supplementary material for: Hidden Xyloglucan Architecture of the Pollen Intine in Gagea lutea Revealed by Sequential Enzymatic Unmasking
Source: Biology (Basel). 2026 Jan 28;15(3):243. doi: 10.3390/biology15030243 (PMC12896889; doi:10.3390/biology15030243)
Supplement: Supplementary file 1 [file biology-15-00243-s001.zip › biology-4072491-supplementary.pdf]

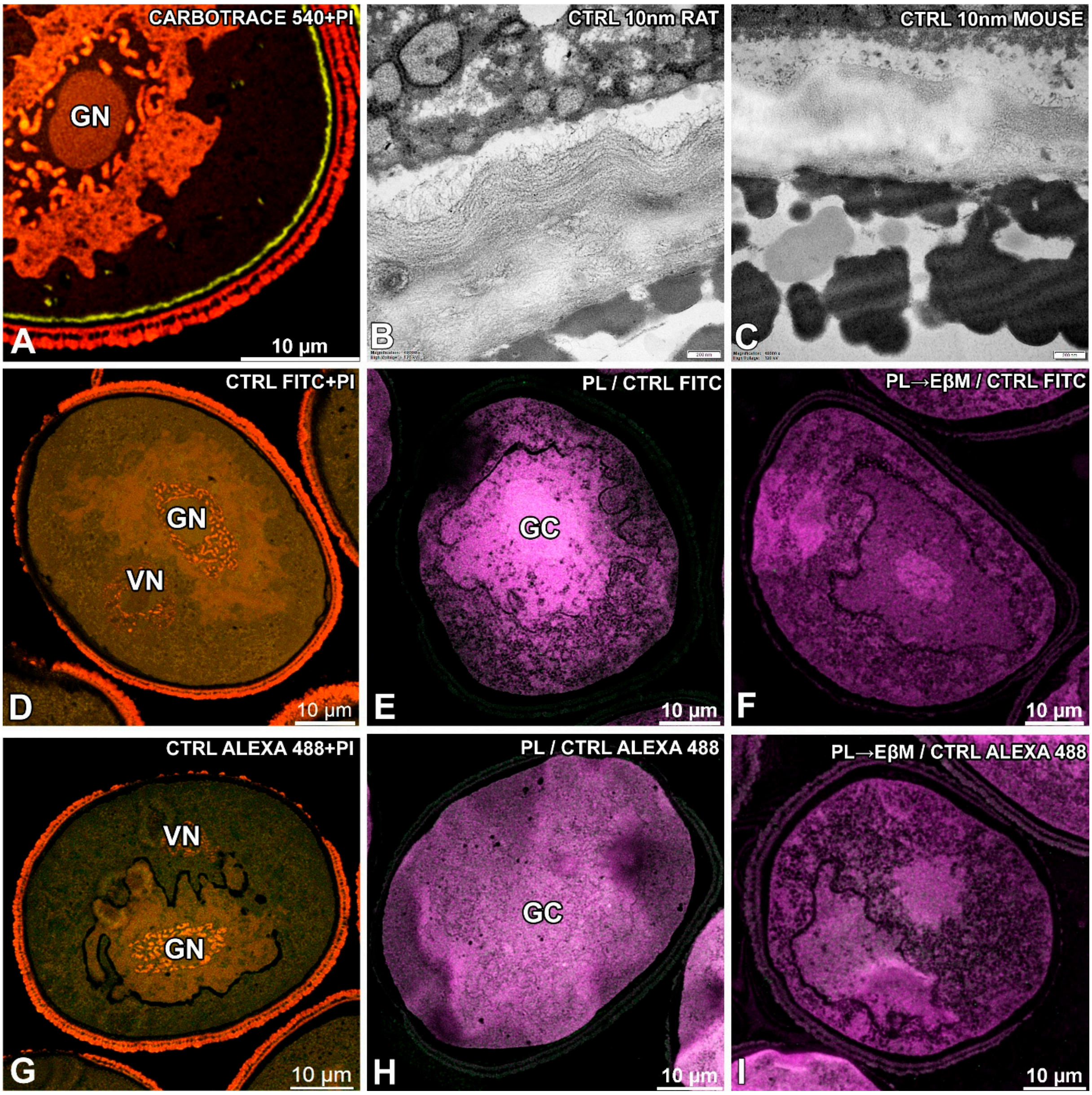

Figure S1. Control reactions for labelling and enzymatic digestion assays in mature pollen grains of Gagea lutea. (A) Counterstaining with Carbotrace 540 together with propidium iodide (PI). (B, C) Negative controls for immunogold TEM using 10 nm gold-labelled secondary antibodies raised against rat (B) or mouse (C) (no primary antibody). (D–F) Negative controls for immunofluorescence using FITC-conjugated secondary antibody (no primary antibody) in untreated sections (D) and after enzymatic digestion with pectate lyase (PL) (E) or sequential pectate lyase followed by mannanase (PL→EβM) (F). (G–I) Negative controls for immunofluorescence using Alexa Fluor 488-conjugated secondary antibody (no primary antibody) in untreated sections (G) and after PL (H) or PL→EβM (I). PI fluorescence is shown in red; magenta indicates autofluorescence. (GC) – generative cell; (GN) – generative nucleus; (VN) – vegetative nucleus. Scale bars: 10 µm (A, D–I); 200 nm (B, C).

Table S1. Corrected mean fluorescence intensity (MFI) measured in the intine ROI. Fixed factors: pool (2 levels), antibody (LM15, LM24, LM25, CCRC-M48) and treatment

(untreated, pectate lyase (PL), PL followed by endo-β-mannanase (PL→EβM)). Statistica v13.3 (TIBCO).

	effect SS	effect Df	effect MS	F	p-level
**Run**	2238	1	2238	2,503	0,115067
**Antibody**	401047	3	133682	149,502	0,000000
**Treatment**	138246	2	69123	77,303	0,000000
**Run*Antibody**	9441	3	3147	3,519	0,015945
**Run*Traatment**	7097	2	3548	3,968	0,020302
**Antibody*Treatment**	25217	6	4203	4,700	0,000161
**Run*Antibody*Treatment**	8783	6	1464	1,637	0,138156
**Error (residual)**	193144	216	894		

Table S2. Post hoc multiple comparisons (Tukey's HSD).

**LM15**	**untreated**											
	**PL**	0,293848										
	**PL→EβM**	0,000018	0,058447									
**LM24**	**untreated**	0,000020	0,000018	0,000018								
	**PL**	0,000287	0,000018	0,000018	0,999113							
	**PL→EβM**	0,987860	0,970954	0,000306	0,000018	0,000018						
**LM25**	**untreated**	0,064062	0,000018	0,000018	0,471481	0,963047	0,000665					
	**PL**	0,640018	0,999998	0,010976	0,000018	0,000018	0,999437	0,000026				
	**PL→EβM**	0,000018	0,017800	1,000000	0,000018	0,000018	0,000064	0,000018	0,002655			
**CCRC-M48**	**untreated**	0,000018	0,000018	0,000018	0,002664	0,000055	0,000018	0,000018	0,000018	0,000018		
	**PL**	0,000018	0,000018	0,000018	0,226487	0,016568	0,000018	0,000051	0,000018	0,000018	0,967402	
	**PL→EβM**	0,000018	0,000018	0,000018	0,989819	0,628092	0,000018	0,023818	0,000018	0,000018	0,144472	0,938562
